# Haematological malignancies in systemic sclerosis: a population-based nationwide register study

**DOI:** 10.1136/rmdopen-2025-005873

**Published:** 2025-11-11

**Authors:** Karin Gunnarsson, Luigi Annicchiarico, Anna Ravn Landtblom, Fredrik Baecklund, Kristofer Andréasson, Marie Holmqvist

**Affiliations:** 1 Division of Clinical Epidemiology, Department of Medicine Solna, Karolinska Institutet, Stockholm, Sweden; 2 Medical Unit Gastroenterology, Dermatology and Rheumatology, Karolinska University Hospital, Stockholm, Sweden; 3 Department of Clinical Sciences and Community Health, Biometry and Epidemiology ‘GA Maccacaro’, University of Milan, Milan, Italy; 4 Center for Hematology and Regenerative Medicine, Department of Medicine Huddinge, Karolinska Institutet, Stockholm, Sweden; 5 Department of Hematology, Karolinska University Hospital, Karolinska Institutet, Stockholm, Sweden; 6 Department of Pediatric Oncology, Karolinska University Hospital, Karolinska Institutet, Stockholm, Sweden; 7 Rare Diseases Unit, Department of Molecular Medicine and Surgery, Karolinska Institutet, Stockholm, Sweden; 8 Department of Rheumatology, Lund University, Lund, Sweden

**Keywords:** Scleroderma, Systemic, Hematology, Epidemiology, B-Lymphocytes

## Abstract

**Objectives:**

(1) To assess the risk of haematological malignancies in individuals with systemic sclerosis (SSc) compared with individuals without SSc; (2) to explore how the risk varies across groups stratified by sex and age; and (3) to determine when these malignancies present in relation to SSc diagnosis in a population-based setting.

**Methods:**

We performed a nationwide cohort study using high-quality administrative healthcare registers covering virtually all Swedish residents. All individuals with SSc diagnosed during 2004–2019 and matched general population comparators were included. We identified all haematological malignancies in the study population using the Swedish Cancer Register and estimated the incidence rates using Poisson regression and the HRs using flexible parametric models. We stratified by sex and age and explored the incidence over time since SSc diagnosis.

**Results:**

We observed 1720 individuals with incident SSc and 16 983 comparators for 11 480 and 131 021 person-years, respectively. Individuals with SSc had a higher risk of haematological malignancies compared with individuals without SSc (HR 2.2, 95% CI 1.4 to 3.1), especially B-cell malignancies (HR 3.0, 95% CI 1.7 to 4.8). The incidence rate and the HR were highest in men. The SSc over-representation of haematological malignancies was most evident in individuals aged 18–49 years at SSc diagnosis. Myeloid malignancies presented around SSc diagnosis (median 0.1 (IQR 8.2) years after SSc diagnosis) while lymphoid malignancies presented a few years later (median 3.1 (IQR 9.5)).

**Conclusion:**

Individuals with SSc are afflicted by an increased risk of haematological malignancies, especially B-cell malignancies. The risk is highest in men. Myeloid malignancies tend to present closer to SSc diagnosis than lymphoid malignancies.

**WHAT IS ALREADY KNOWN ON THIS TOPIC**

- We know that individuals with systemic sclerosis (SSc) are afflicted by an increased risk of haematological malignancies.

**WHAT THIS STUDY ADDS**

- Men exhibit a higher risk than women and the association is strongest in younger individuals (aged 18–49).
- The risk is most pronounced for lymphoid malignancies, particularly B-cell malignancies, which typically present a few years after SSc diagnosis, whereas myeloid malignancies tend to debut in closer temporal proximity to SSc diagnosis.

**HOW THIS STUDY MIGHT AFFECT RESEARCH, PRACTICE OR POLICY**

- These results could assist clinicians in identifying individuals with SSc who require heightened vigilance for haematological malignancies and in developing more specific cancer screening algorithms.
- Future studies could further investigate the distinct mechanisms underlying the association between various subgroups of haematological malignancies and SSc.

## Introduction

 Systemic sclerosis (SSc) is a rare systemic rheumatic disease (SRD) characterised by fibrosis, vasculopathy and autoimmunity. SSc carries one of the highest mortality rates of all SRDs^1^ which is attributed to disease-related causes, mainly lung and heart involvement,^2^ and comorbidities, such as cancer, cardiovascular disease and infections. Among these comorbidities, cancer is the most common cause of death in individuals with SSc.^3^ Individuals with SSc are afflicted by a 40–120% increased risk of cancer overall after diagnosis when compared with individuals without SSc,^4^,^9^ and the risk of haematological malignancies is increased by 50–150%.^4^,^610 11^

Several meta-analyses on cancer and SSc have been published,^4 5 12^ but most of the included studies lacked a comparison group for contextualisation, and few assessed the temporal relationship between malignancies and SSc. Studies on the specific risks of non-Hodgkin’s lymphomas, leukaemias, multiple myeloma and myelodysplastic syndrome (MDS) are scarce but risk estimates have been presented on SSc and different types of solid and haematological malignancies,^47 9^,^11^ and on multiple myeloma in hospitalised individuals with autoimmune diseases.^13 14^ Previous studies on myeloproliferative neoplasms (MPNs) and SSc have been a case series^15^ and a study that found no association.^16^ Recent studies focusing specifically on haematological malignancies and SSc have either included only hospital-based comparators,^10^ been case series^15^ or employed a registry-based cohort that was not population-based,^11^ so the risks have not yet been established in a population-based setting, including general population comparators. Hence, there is a need for updated population-based studies with matched comparators to obtain robust and contemporary data on the relationship of SSc and haematological malignancies.

We therefore set out to obtain detailed data on which haematological malignancies individuals with SSc are at increased risk of, and how this risk varies over time since SSc diagnosis, using Swedish administrative healthcare registers covering virtually all residents. We also wanted to explore how this risk differs in groups stratified by sex and age at SSc diagnosis to inform clinical decision-making.

## Methods

### Study design and setting

In Sweden, healthcare is tax funded and equally accessible for virtually all residents. Within this setting, we have performed a population-based matched cohort study during 2004–2020. Data for the study have been extracted from nationwide administrative registers containing information from the healthcare system covering the entire population of Sweden.

### Data sources

The National Patient Register (NPR) holds nationwide information on hospitalisations since 1987 and outpatient visits since 2001, including date of hospitalisation/visit and main and ancillary diagnoses as International Classification of Disease (ICD) codes.^17^ The Swedish Cancer Register (SCR) started in 1958, and since then reporting information on all primary malignancies is mandatory for pathologists and clinicians and is regulated by Swedish law. Reported information includes date of diagnosis, ICD code and, since 1993, the tumour type coded according to the Systematized Nomenclature of Medicine (SNOMED). The SCR is estimated to capture around 95% of all cancers diagnosed within public and private care.^18^ The 5% missingness is mostly due to under-reporting of aggressive tumours of internal organs in older individuals, who die before they are properly diagnosed and entered in the register.^19^ The completeness and accuracy of haematological malignancies in the SCR are also reported to be high.^20^ The Cause of Death Register (CDR) contains information on causes of death since 1952. The underlying cause is registered in 98% of all deaths since 1997.^21^ The Total Population Register (TPR) contains demographic data on virtually all residing in Sweden.^22^ All data in the national registers are collected prospectively. Each individual in Sweden has a unique identification number.^23^ We used this number when linking information from different registers on an individual level.

### Study population, exposure and additional covariates

We identified individuals with incident SSc in Sweden between 2004 and 2019 using the NPR as described in a previous register-based study estimating the prevalence and incidence of SSc in Sweden.^24^ To be classified as incident SSc, we required two outpatient visits or hospitalisations with an ICD-10 code indicating SSc (M34.0, M34.1, M34.8 or M34.9) as main diagnosis within 12 months. At least one of the visits/hospitalisations was required to be in a rheumatology or internal medicine clinic to minimise the risk of misclassification. To ensure that we captured incident cases, we required all included individuals to not have any visits listing SSc as main or contributory diagnosis prior to the first disease-defining visit. Individuals had to live in Sweden and be at least 18 years old at the second visit indicating SSc (the index date).

We identified up to 10 general population comparators (minimum 3, median 10) from the TPR requiring these individuals to be free from SSc, living in Sweden and to be alive at the index date. The comparators were matched to the individuals with SSc on sex, birth year and residential area but were otherwise selected as a random sample from the general population. The same index date was assigned to the comparators as their matched individual with SSc. Two individuals with SSc were excluded due to missing data on residential area.

### Outcome and follow-up

**Figure 1:**
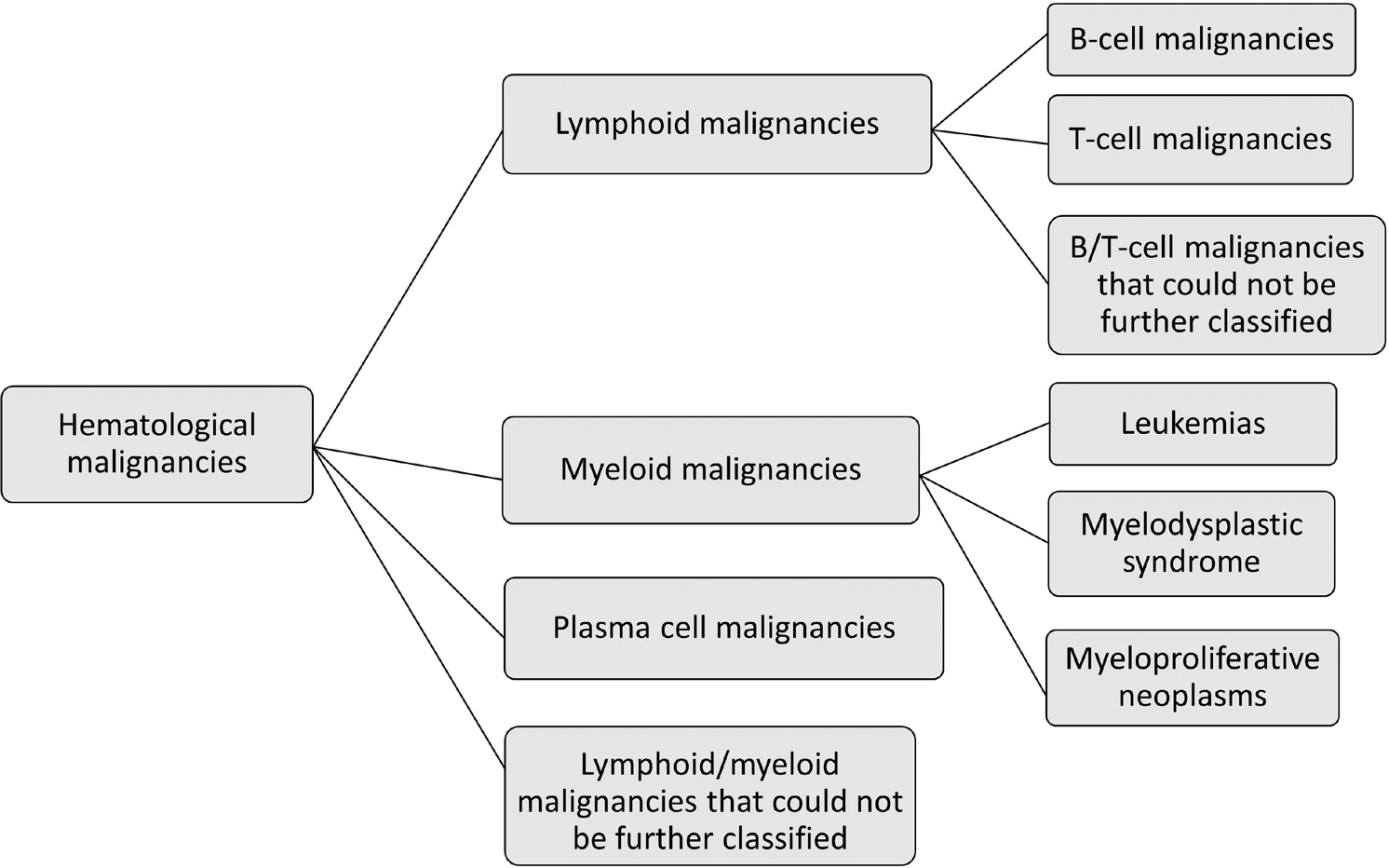
Schematic presentation of the classification of haematological malignancies in clinically relevant subgroups. Some malignancies are included in more than one outcome definition, for example, observations of B-cell malignancies also contribute to the group of lymphoid malignancies.

To assess the primary outcome, all primary haematological malignancies were defined by ICD-7, ICD-8, ICD-9 or ICD-10 and SNOMED codes^25^ primarily from the SCR. We used the code providing the most detailed diagnosis; ICD-10 and SNOMED codes had first priority, ICD-9 second, ICD-8 third and ICD-7 code fourth priority. For a complete list of the codes and how they were used to classify outcomes during different calendar periods, see online supplemental tables S1–S3. Some malignancies are included in more than one outcome definition (figure 1). Information on death from haematological malignancies was extracted from the CDR. Date of death was then used to approximate the date of malignancy diagnosis.

To identify and classify MPNs, we used data from the NPR in addition to the SCR. For data from the NPR, we required two visits on two separate occasions for MPNs. Only the first ever MPN was considered.

All the outcomes were analysed separately.

The start of follow-up was the index date. The end of follow-up was concluded by having the outcome of interest, death, emigration or the end of study period, set to 31 December 2020.

### Statistical analysis

We described the study population by SSc status at index date using means and SDs, medians and IQRs, frequencies and percentages as appropriate.

In our main analyses, SSc was the exposure. The first ever visit/registration indicating the outcome of interest (haematological malignancies overall and by subgroup) after the index date was considered as a time to event variable. Individuals who had the specific outcome of interest before the start of follow-up did not contribute person-time or events in that specific analysis. We did not censor at solid malignancies diagnosed after the index date, as the individual was still considered to be at risk of haematological malignancies. Models in our main analyses were adjusted by the known confounders sex and age. Unadjusted incidence rates with 95% CIs were estimated using Poisson models, and the rate difference was calculated for each outcome. We also stratified our analyses by sex, age groups defined by distribution of age per quartiles in the entire study population and subgroups of haematological malignancies.

Flexible parametric models^26^ were used to assess the association between SSc and haematological malignancies over time, modelling splines with df=3. HRs were estimated throughout follow-up as well as 1, 5 and 10 years after index date. We also estimated 1, 5 and 10 years’ cumulative incidence of haematological malignancies overall, in individuals with SSc and comparators, respectively, as a measure of absolute risk.

We described the number (% of all) of malignancies in individuals with SSc and comparators, respectively, in the time around the index date (±5 years) as well as more than 5 years after index date, and how these were distributed over time. We assessed the association between SSc and haematological malignancies presenting within ±5 years from the index date using conditional logistic regression models.

The analyses were conducted using SAS V.9.4 (SAS Institute) and R V.4.4,^27^ with packages rstpm2,^28^ dplyr,^29^ survival,^30^ forest plot^31^ and ggplot.^32^

### Sensitivity analyses

As a sensitivity analysis, we excluded patients with a malignancy of any type (solid or haematological) occurring before the index date from the data set. Additionally, we conducted a sensitivity analysis in which individuals who developed solid cancer after the index date were censored at the time of solid cancer diagnosis. Individuals with a solid cancer prior to the index date were excluded from this analysis.

Furthermore, we conducted a sensitivity analysis in which individuals with an ICD-10 code indicating Sjögren’s disease (M35.0, M35.0A, M35.0B) were censored at the time of Sjögren’s disease. Individuals with an ICD-10 code indicating Sjögren’s disease prior to the index date were excluded. This analysis was conducted due to the documented association between SSc and Sjögren’s disease, another condition known to be linked with haematological malignancies.

Finally, we performed the conditional regression analysis of the association between SSc and cancer within 3 years from the index date (instead of 5 years).

### Additional analyses

We analysed if patients with acute myeloid leukaemia (AML) or MDS had any history of malignancy (solid or haematological), previous to the AML/MDS, as these types of malignancies are considered secondary if a patient has received radiotherapy or chemotherapy against a previous malignancy.

## Results

We identified 1720 individuals with incident SSc and 16 983 matched general population comparators. 81% of both individuals with SSc and comparators were women and the mean age at index date was 59 years (table 1).

**Table 1:** Baseline characteristics of the study population of individuals with incident systemic sclerosis during 2004–2019 and their matched general population comparators

	Individuals with systemic sclerosis (n=1720)	General population comparators (n=16 983)
Sex, women, n (%)	1388 (81)	13 704 (81)
Age at index date, mean (SD)	58.8 (14.9)	58.7 (14.9)
Age in years at index date, in categories, n (%)		
18–49	466 (27)	4670 (27)
50–59	375 (22)	3696 (22)
60–69	445 (26)	4383 (26)
>70	434 (25)	4234 (25)

n denotes number of observations.

### Occurrence of haematological malignancies after index date

The 1720 individuals with SSc were observed during 11 480 person-years (median duration 5.8 years (IQR 6.8)), and the 16 983 comparators were observed during 131 021 person-years (median duration 7.2 years (IQR 7.3)). During follow-up, we identified first-time haematological malignancies in 27 individuals with SSc (1.6%) and in 154 comparators (0.9%). This corresponded to a crude incidence rate for haematological malignancies overall in individuals with SSc of 2.4/1000 person-years (95% CI 1.6 to 3.4), and 1.2/1000 person-years (95% CI 1.0 to 1.4) in the comparators. The crude rate difference was 1.2/1000 person-years (95% CI 0.3 to 2.1) (table 2).

**Table 2:** Haematological malignancies presenting after the index date

	Individuals with systemic sclerosis			Matched comparators			Crude rate difference (95% CI)*1000 person-years	HR overall (95% CI)	HR at 1 year	HR at 5 years	HR at 10 years
	Events, n	Person-years	Crude IR (95% CI)*1000 person-years	Events, n	Person-years	Crude IR (95% CI)*1000 person-years					
Overall	27	11 480	2.4 (1.6 to 3.4)	154	131 021	1.2 (1.0 to 1.4)	1.2 (0.3 to 2.1)	2.2 (1.4 to 3.1)	1.7 (0.8–3.5)	2.1 (1.4–3.3)	2.6 (1.4–4.8)
Men	8	2207	3.6 (1.8 to 7.2)	34	26 159	1.3 (0.9 to 1.8)	2.3 (−0.2 to 4.9)	3.1 (1.4 to 5.7)	3.4 (0.8–14.2)	2.5 (1.1–5.8)	3.6 (1.0–12.6)
Women	19	9272	2.1 (1.3 to 3.2)	120	104 861	1.1 (1.0 to 1.4)	0.9 (0.0 to 1.8)	1.9 (1.2 to 2.9)	1.4 (0.6–3.4)	2.0 (1.2–3.2)	2.4 (1.2–4.9)
Age at index date, in categories											
18–49	8	3638	2.2 (1.1 to 4.4)	11	37 883	0.3 (0.2 to 0.5)	1.9 (0.4 to 3.4)	7.7 (3.5 to 14.3)			
50–59	6	2879	2.1 (0.9 to 4.6)	32	30 933	1.0 (0.7 to 1.5)	1.1 (−0.7 to 2.8)	2.0 (0.8 to 4.1)			
60–69	3	2951	1.0 (0.3 to 3.1)	51	35 301	1.4 (1.1 to 1.9)	−0.4 (−1.6 to 0.8)	0.7 (0.2 to 1.9)			
>70	10	2010	5.0 (2.7 to 9.2)	60	26 902	2.2 (1.7 to 2.9)	2.7 (−0.4 to 5.9)	2.3 (1.1 to 4.0)			
Subgroups of haematological malignancies											
Lymphoid malignancies	15	11 536	1.3 (0.8 to 2.2)	75	131 624	0.6 (0.4 to 0.7)	0.7 (0.1 to 1.4)	2.5 (1.4 to 3.9)	1.7 (0.6–5.1)	2.0 (1.1–3.7)	3.6 (1.7–7.8)
B-cell malignancies	15	11 549	1.3 (0.8 to 2.1)	62	131 808	0.5 (0.4 to 0.6)	0.8 (0.2 to 1.5)	3.0 (1.7 to 4.8)	2.3 (0.8–6.8)	2.5 (1.4–4.6)	4.1 (1.9–8.9)
Myeloid malignancies	8	11 557	0.7 (0.3 to 1.4)	57	131 790	0.4 (0.3 to 0.6)	0.3 (−0.2 to 0.7)	1.7 (0.8 to 3.2)	2.0 (0.6–6.5)	1.7 (0.8–3.7)	1.5 (0.4–4.9)
Plasma cell malignancies	5	11 601	0.4 (0.2 to 1.0)	25	132 165	0.2 (0.1 to 0.3)	0.2 (−0.1 to 0.6)	2.4 (0.9 to 5.2)			

Crude IRs and rate differences of haematological malignancies overall and by subgroups, in individuals with systemic sclerosis and matched general population comparators. HRs for haematological malignancies comparing individuals with systemic sclerosis and comparators are adjusted for age and sex, and further stratified by sex, age categories and subgroups of haematological malignancies.

n denotes number of observations.

IR, incidence rate.

**Figure 2:**
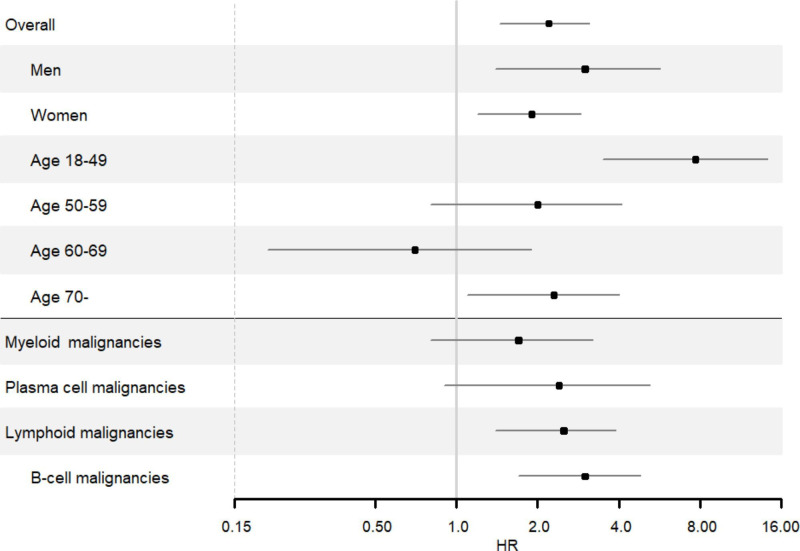
HRs and 95% CIs of haematological malignancies in individuals with systemic sclerosis versus general population comparators, overall and stratified by sex, age at index date and subgroups of haematological malignancies.

This corresponded to a HR of 2.2 (95% CI 1.4 to 3.5) for haematological malignancies overall in individuals with SSc compared with the general population comparators. In men, the corresponding HR was 3.1, and in women 1.9. The association was especially pronounced in individuals aged 18–49 years at index date (HR 7.7, 95% CI 3.5 to 14.3). The HRs for lymphoid, plasma cell, myeloid and B-cell malignancies were 2.5 (95% CI 1.4 to 3.9), 2.4 (95% CI 0.9 to 5.2), 1.7 (95% CI 0.8 to 3.2) and 3.0 (95% CI 1.7 to 4.8), respectively (figure 2).

The number of events in the analyses of subgroups of haematological malignancies does not match the total number of events in the haematological malignancies’ overall analysis. This discrepancy arises from our method of classifying subgroups, in which individuals with a history of the outcome of interest did not contribute person-time or events in that specific analysis. One individual with a B-cell malignancy prior to the index date was excluded from the analysis of haematological malignancies overall and from the B-cell malignancy subgroup. However, this same individual presented with a malignancy which was included as a case in the plasma cell malignancy analysis, as the individual had no history of that specific outcome. Furthermore, the subgroups are not mutually exclusive; therefore, the cases in the T-cell and B-cell malignancy groups are also included in the lymphoid malignancy group.

Among individuals with SSc, MPNs were the most frequent myeloid malignancies (n=9), followed by AML (n=2). The most common MPN among individuals with SSc was essential thrombocythaemia (ET) (n=5). All lymphoid malignancies in the individuals with SSc were of B-cell origin, while among the comparators, six T-cell malignancies were identified. The most common B-cell lymphoma was diffuse large B-cell lymphoma (online supplemental table S4).

### Temporal relationship of SSc and haematological malignancies

**Figure 3:**
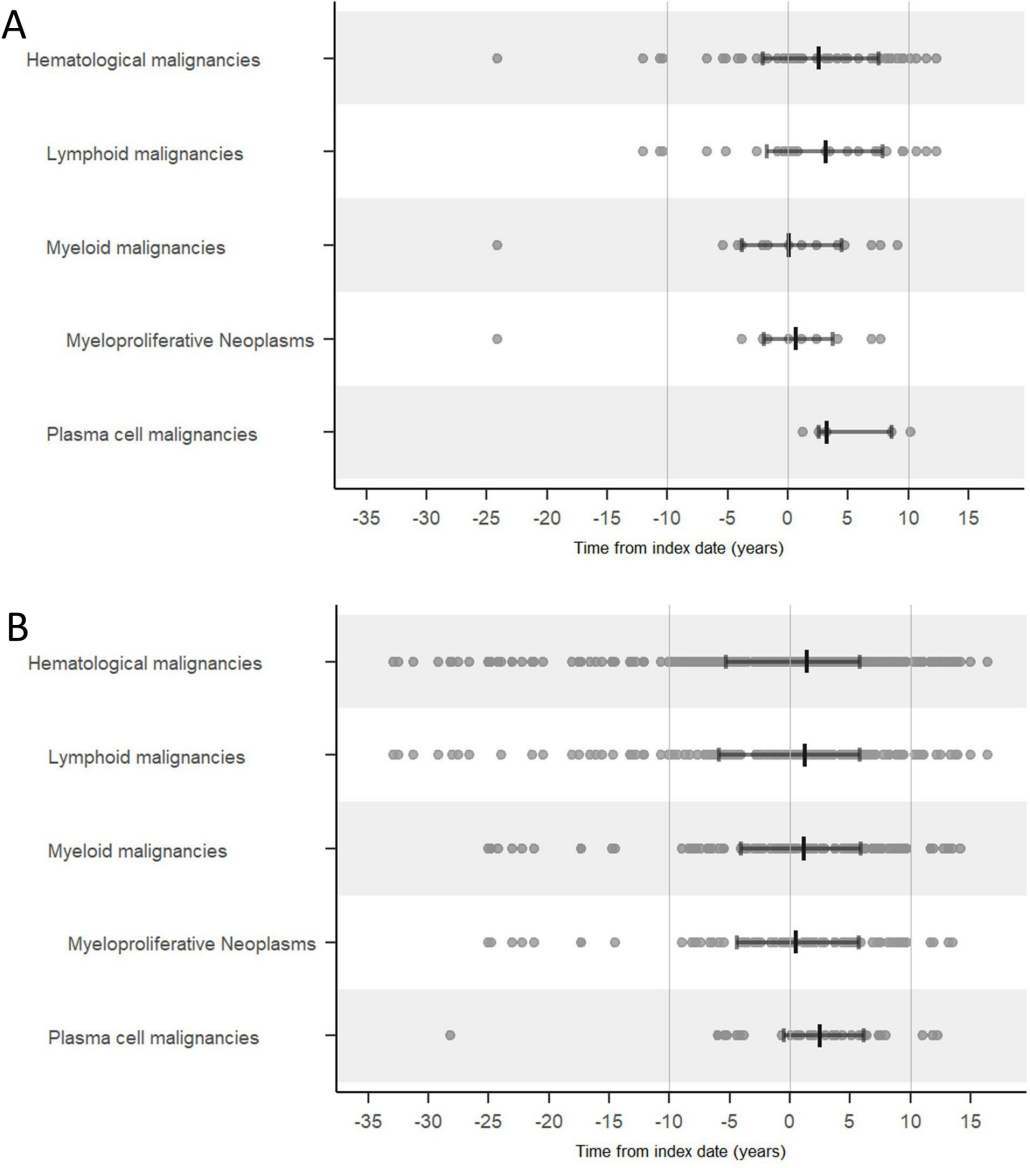
Temporal relationship between systemic sclerosis diagnosis (the index date) and the presentation of haematological malignancies, overall and divided by subgroups, in individuals with systemic sclerosis (**A**). The corresponding malignancies in comparators (**B**) are displayed for comparison. Time (years) from the index date (time=0) is plotted for each detected malignancy, median and quartiles marked.

The majority of the haematological malignancies overall were diagnosed a few years after the index date (median 2.6 years (IQR 9.6)). Myeloid malignancies overall and MPNs presented closer to the index date (median 0.1 years (IQR 8.2) and 0.6 years (IQR 5.7), respectively, after the index date), while lymphoid and plasma cell malignancies presented several years after the index date (median 3.1 (IQR 9.5) and 3.2 (IQR 6.0), respectively) and continued to present during the 10 years after the index date (figure 3).

**Figure 4:**
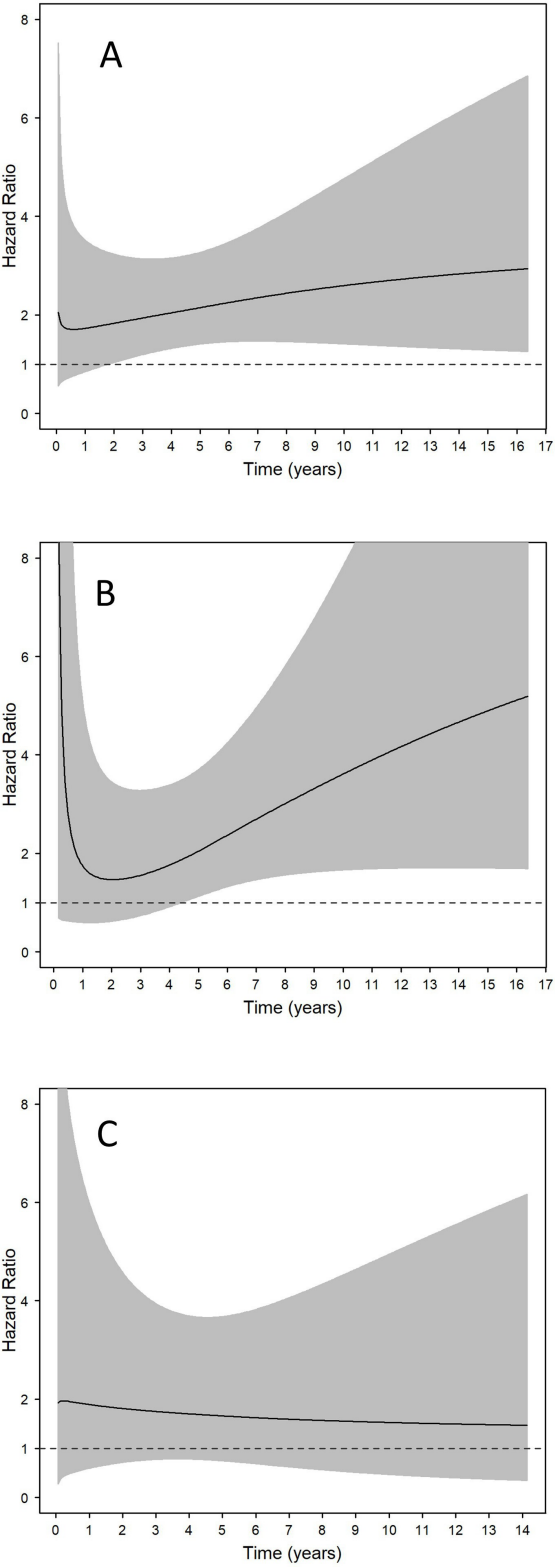
HRs for haematological malignancies overall (**A**) in individuals with systemic sclerosis versus matched comparators, variations over time since systemic sclerosis diagnosis (index date, time=0). HRs over time for lymphoid malignancies (**B**) and myeloid malignancies (**C**). 95% CI is represented by the grey area.

We observed an increasing HR for haematological malignancies overall and lymphoid neoplasms over the 10 years following the index date and a tendency of decreasing HR for myeloid neoplasms over 10 years (table 2, figure 4). The cumulative incidence for haematological malignancies overall at 1, 5 and 10 years after the index date was 0.2%, 1.0% and 2.7%, respectively, in individuals with SSc and 0.1%, 0.5% and 1.2%, respectively, in comparators.

We observed an association of haematological malignancies and SSc (OR 1.8, 95% CI 1.1 to 2.9) in the period around the index date (±5 years). The association during this period was somewhat stronger for myeloid malignancies (OR 2.1, 95% CI 1.0 to 4.4) than for lymphoid malignancies (OR 1.6, 95% CI 0.8 to 3.3) and plasma cell malignancies (OR 1.7, 95% CI 0.5 to 5.8), but CIs were overlapping.

### Sensitivity analyses

After excluding individuals with a history of malignancy, we still observed an association between SSc and haematological malignancies overall (HR 2.3, 95% CI 1.5 to 3.3; online supplemental table S5). Moreover, the estimates from the stratified groups in this sensitivity analysis were consistent with the findings from the primary analysis (table 2). The results from this sensitivity analysis did not change our interpretation of the main findings regarding the occurrence of haematological malignancies after the index date.

Neither the analysis censoring individuals with solid cancer after the index date yielded estimates that altered our interpretation of our main findings. If anything, the association between SSc and haematological malignancies appeared even stronger, except in the 18–49 years’ age category, where the association was slightly weaker (online supplemental table S6).

The estimates from the analysis excluding individuals with an ICD-10 code indicating Sjögren’s disease did not alter the interpretation of our main findings (online supplemental table S7).

In the sensitivity analysis assessing the association of SSc and haematological malignancies ±3 years from the index date, the OR was 1.6 (95% CI 0.8 to 2.9). Neither this changed our interpretation of the primary analysis.

### Additional analyses

We identified 15 individuals with AML. None of them had a history of malignancy before AML. We identified 12 individuals with MDS. Among these, two had a history of MPN and one of squamous cell carcinoma prior to the MDS diagnosis.

## Discussion

In this population-based nationwide register study, we report a significantly increased risk of haematological malignancies, after SSc diagnosis, in individuals with incident SSc compared with age and sex-matched individuals without SSc. This increased risk was most prominent for B-cell malignancies. The main results of the present study are in line with results from previous studies^4^,^610 11^ which strengthen the interpretation that patients with SSc indeed are at increased risk of haematological malignancies.

A noteworthy observation was the pronounced over-representation of haematological malignancies in individuals with SSc diagnosed at 18–49 years. This is likely partly due to the low background risk of haematological malignancies in this age group in individuals without SSc. Notably, the incidence rate for individuals with SSc in this young age group was comparable to that of individuals >70 years without SSc, which is striking given the higher general prevalence of haematological malignancies in older individuals. In our study, the highest incidence rate among individuals with SSc was in those with onset >70 years, consistent with the general population’s trend of increasing haematological malignancies with age.^33^ This finding also aligns with a recent Spanish register study^6^ and a large Australian cohort study,^9^ where high age at SSc diagnosis was associated with increased risk of haematological malignancies among individuals with SSc.

The incidence rate of haematological cancer was higher in men than in women, both in individuals with SSc and comparators, with the highest rate in men with SSc. This contrasts with the Spanish study,^6^ which did not confirm an increased risk in men with SSc compared with the expected incidence. Differences in background malignancy incidence rates and potential confounders between the Spanish and Swedish populations may explain this discrepancy. An Italian case series^15^ and a meta-analysis^4^ also indicated higher standardised incidence ratios for haematological malignancies in men with SSc, similar to findings in a Danish register study.^34^ This is in line with what we see in the general population where haematological malignancies in general are more common in men.^35^ Our results confirm the increased relative and absolute risk of haematological malignancies in men with SSc.

We did not observe a statistically significant increased risk of myeloid or plasma cell malignancies after index date in individuals with SSc, despite previous studies suggesting such a risk.^7 11 13 14^ Our results are in line with a study that could not confirm any increased risk of multiple myeloma in individuals with SSc,^10^ but our point estimates align with the previous findings, suggesting an association between SSc and myeloid and plasma cell malignancies. The lack of statistically significant results in this analysis may be attributable to insufficient statistical power. We noted a somewhat increased occurrence of MPNs during follow-up, primarily presenting around SSc onset, with ET being the most common MPN, contrasting with the Italian case series findings.^15^

All lymphoid malignancies in our study were of B-cell origin, with diffuse large B-cell lymphoma being the most common subtype, similar to the Italian case series findings.^15^ Autoreactive and dysregulated B cells are central in SSc pathogenesis and are found in affected organs like the skin and the lungs.^36^ Also, B-cell depleting therapies, like inhibitors of CD19, CD20 or CAR-T (chimeric antigen receptor T-cell) therapy targeting CD19, seem effective in SSc.^37^ It has been hypothesised that autoreactive B cells may be susceptible to malignant transformation.^38^ Other potential links between SSc and haematological malignancies include chronic inflammation,^39^ treatment-related malignancies,^40^ shared genetic susceptibility or long-standing viral infections in immunosuppressed individuals.

When applying an alternative time frame, within ±5 years of SSc diagnosis, an association between SSc and haematological malignancies was also observed. In this analysis, we observed a tendency towards a stronger association in myeloid malignancies compared with lymphoid and plasma cell malignancies, although with overlapping CIs. Myeloid malignancies mainly presented around the index date, while lymphoid neoplasms appeared a few years later, with high occurrence throughout the 10 years following SSc onset.

In a case series, most haematological malignancies were diagnosed within 5 years from SSc diagnosis.^15^ Lymphomas have both been described to present early, mainly during the first 4 years after SSc diagnosis,^41^ and later. In an Australian cohort study, non-Hodgkin’s lymphomas tended to present mainly >5 years from SSc diagnosis; however, statistical significance was not reached in this analysis.^9^ In another Australian study, all haematological malignancies occurred at or after SSc onset.^42^ When it comes to solid malignancies in SSc, some types of cancer have been identified as early-onset cancers presenting ±5 years around SSc diagnosis, for example, breast and melanoma skin cancer,^9^ while others present later.^43^ This difference in temporal relationship suggests different mechanisms, whereby cancer may induce autoimmunity in early-onset cancers.^44^ This could possibly be one of the mechanisms triggering the onset of myeloid malignancies appearing around SSc onset. Moreover, myeloid cells have been reported to play a central role in fibrogenesis and are dysregulated in SSc,^45^ potentially creating a permissive environment for malignant transformation of this cell lineage.

In rheumatoid arthritis (RA), an increased risk of lymphomas, likely associated with long-standing inflammation, has been identified, with lymphomas debuting about 10 years after RA onset.^46^ In Sjögren’s disease, lymphomas present at a median of 7 years after diagnosis.^47^ The median time from SSc onset to haematological malignancies in our study was shorter than in RA and Sjögren’s, suggesting different mechanisms. Diagnostic delay in SSc may also contribute to this difference.

Strengths of our study include the use of high-quality administrative healthcare registers with nationwide coverage, avoiding selection biases related to specific hospitals or geographical areas. The substantial study size, given the low prevalence of SSc, and the regulated reporting to the SCR ensure high coverage and reliability.^48^ Using matched general population comparators provided contemporary and unselected background risks, avoiding biases from hospitalised individuals without SSc.

Limitations include the lack of information on some possible confounders, such as immunodeficiencies or genetic factors, leading to residual confounding. However, we adjusted for the most important risk factors for lymphomas: age and sex. We also lacked information on clinically relevant features like serological profile and SSc subtype, known to correlate with cancer risk,^42 49^ as these are not always specified in the NPR. This information would aid clinicians in risk stratifying patients.

Another limitation of the present study concerns the absence of an analysis of, or adjustment for, immunosuppressive medications, which may influence the risk of haematological malignancies. This omission is partly due to the limitations of Swedish administrative healthcare data, which only include information on orally or subcutaneously administered drugs dispensed at pharmacies. Consequently, several medications of particular interest, such as rituximab and intravenously administered cyclophosphamide, would not be analysed. Furthermore, most immunosuppressive drugs used in modern antirheumatic treatment are not associated with an increased risk of malignancy. For instance, in RA, observational data suggest that the elevated risk of lymphoma is more likely attributable to the underlying disease itself rather than to the use of specific antirheumatic drugs.^50^ The risk of AML and MDS is increased in individuals who previously have been treated due to a primary malignancy. In our data, none of the individuals diagnosed with AML and only two individuals with MDS had a history of previous primary malignancy.

Primary Sjögren’s disease has been reported to be associated with various haematological malignancies, including lymphomas, Waldenström’s macroglobulinaemia and leukaemias.^51^ The lack of high-quality information and assessment of secondary Sjögren’s disease, which has previously been suggested to confer an even higher risk of non-Hodgkin’s lymphoma than primary Sjögren’s disease alone,^52^ is an additional limitation to our study. We consider that secondary Sjögren’s disease is not reliably coded in the Swedish Patient Register. This conclusion is based on a comparison between the prevalence observed in our study cohort (8.4%) and previously reported prevalence estimates (12.6–29.7%).^52^ However, acknowledging these limitations, we conducted a sensitivity analysis excluding individuals with an ICD-10 code indicating Sjögren’s disease, and the results were consistent with those of the primary analysis.

A shared pathogenesis between premalignant haematological disorders and autoimmunity has been proposed. Notably, the prevalence of clonal haematopoiesis of indeterminate potential (CHIP) has recently been reported to be increased among individuals with SSc.^53^ However, due to the risk of detection bias, we chose not to include an analysis of premalignant conditions like monoclonal gammopathy of undetermined significance or CHIP in the present study. These disorders are often asymptomatic, and blood sampling is more frequent in individuals with SSc than in comparators, particularly around the time of SSc diagnosis, which potentially could infer biased results and incorrect interpretation of data.

In this comprehensive register-based study of nearly all Swedish residents, we confirmed the increased risk of haematological malignancies in individuals with SSc, as described in previous studies. Our findings also suggest differences in the temporal relationship: myeloid malignancies typically present around SSc onset, while lymphoid malignancies appear a few years later. The risk was highest in men, and the over-representation of haematological malignancies was especially pronounced in individuals aged 18–49 years. These results align with previous studies, reinforcing the increased risk of haematological malignancies in individuals with SSc.

This study provides valuable insights for clinicians monitoring patients with SSc at risk of haematological malignancies and may aid in developing targeted cancer screening algorithms. Despite the increased risk, the absolute risk remains low, emphasising the need for selective screening and reassuring patients about their individual risk levels.

## Supplementary material

10.1136/rmdopen-2025-005873 online supplemental file 1

## Data Availability

Data are available upon reasonable request.
