# Supplementary material for: Haematological malignancies in systemic sclerosis: a population-based nationwide register study
Source: RMD Open. 2025 Nov 11;11(4):e005873. doi: 10.1136/rmdopen-2025-005873 (PMC12606508; doi:10.1136/rmdopen-2025-005873)
Supplement: online supplemental file 1 [file rmdopen-11-4-s001.docx]

**Supplementary table S1**

SNOMED and ICD codes used to classify the hematological malignancies into clinically relevant subgroups

| **Subgroups of hematological malignancies** | **Morphologic code ICD-O/3** | **Morphologic code** **ICD-O/2** | **ICD-10** | **ICD-9** | **ICD-8** | **ICD-7** |
| --- | --- | --- | --- | --- | --- | --- |
| **Lymphoid malignancies** | 96903, 96953, 96983, 96893, 959136, 98263, 96873, 982336, 96803, 99403, 96733, 96993, 98363, 959131, 97613, 98353, 96703, 95903, 959133, 96513, 96503, 96633, 97053, 97093, 97163, 97003, 97023, 97013, 97083, 959135 | 96903, 96923, 959036, 98233, 982336, 96833, 99403, 97113,  97613, 96713,  95903, 95913,  98201 & ICDO10=C919, 96523, 96633, 97003, 97001 & ICDO10=C840, 97023, 97013, 971436 | C911, C833,  C859,  C819 | 2041, 2024, 2092,  2001, 2028 | 20220 | 2041, 2002, 2003,  2000,  2001, 2021, 2040, 2043, 2049,  201,  2022 |
| B-cell lymphoma | 96903, 96953, 96983, 96893, 959136, 98263, 96873, 982336, 96803, 99403, 96733, 96993, 98363, 959131, 97613 | 96903, 96923, 959036, 98233, 982336, 96833, 99403, 97113, 97613, 96713 | C911, C833 | 2041, 2024, 2092 |  | 2041, 2002, 2003 |
| T-cell lymphoma | 97053, 97093, 97163, 97003, 97023, 97013, 97083, 959135 | 97003, 97001 & ICDO10=C840, 97023, 97013, 971436 |  |  | 20220 | 2022 |
| B/T-cell lymphoma* | 98353, 96703, 95903, 959133 | 95903, 95913, 98201 & ICDO10=C919 | C859 | 2001,2028 |  | 2000 ,2001, 2021, 2040, 2043, 2049 |
| Hodgkin lymphoma | 96513, 96503, 96633 | 96523, 96633 | C819 |  |  | 201 |
| **Myeloid malignancies** | 98613, 98913, 98333, 98663,  99453 ,99643, 99603, 99613,  99823 ,99833, 99863, 99893, 99803, 99853, 99891, 99933,   99621, 99623,  99611,  99501, 99503,  99601, 99751 | 98913, 98613, 98673, 98253, 98033, 98633, 98683, 99601, 77860,  99811, 99831, 99891, 99611 & ICD10=D471 | C920, C923,  D473,  D474,  D459,  D471 | 2050,  2051 ,2080,   238H, 2079,  289W,  238E/238.4,  2091,  238H | 20500,  20900,   207.1-9,  209,  208 | 2050, 2060,  2051,  2079,  209,  208 |
| Acute myeloid leukemia | 98613, 98913, 98333, 98663 | 98913, 98613, 98673, 98253 | C920, C923 | 2050 | 20500 | 2050, 2060 |
| Chronic myeloid leukemia | 99453 ,99643, 99603, 99613 | 98033, 98633, 98683, 99601 |  | 2051 ,2080 | 20900 | 2051 |
| Myelodysplastic syndrome | 99823 ,99833, 99863 ,99893, 99803 ,99853, 99891, 99933 | 9811, 99831, 99891, 99611 & ICD10=D471 |  |  |  |  |
| **Lymph/myeloid malignancies*** | 98013 | 98013, 98003 | C950 | 2089 | 20790 | 2070, 2079 |
| **Plasma cell malignancies** | 97323, 97313 | 97323 | C900 |  | 20300 | 203, 2030 |

*The Lymph/myeloid malignancies group gathers malignancies of uncertain nature (lymphoid or myeloid precursors involved) which could not be further classified. Similarly, the B/T cell lymphoma group contains lymphomas with uncertain T or B cell classification.

If a SNOMED code in combination with an ICD code were required for classification these are marked with “&”.

Only primary malignancies should be reported to the SCR but if the same type of cancer was registered in the same individual more than once within three months, we considered this a duplicate registration and only the first registration was used in our analyses.

**Supplementary table S2**

SNOMED and ICD codes used for further subclassification of hematological malignancies in the B cell lymphoma group, which are of particular interest in individuals with systemic sclerosis

| **Subgroups of B cell lymphomas** | **Morphologic code ICD-O/3** | **Morphologic code**  **ICD-O/2** | **ICD-10** | **ICD-9** | **ICD-7** |
| --- | --- | --- | --- | --- | --- |
| Follicular lymphoma | 96903, 96953, 96983 | 96903, 96923 |  |  |  |
| Marginal zone lymphoma splenic | 96893 |  |  |  |  |
| B-cell lymphoma not otherwise specified | 959136 | 959036 |  |  |  |
| Burkitt lymphoma | 98263, 96873 |  |  |  |  |
| Chronic lymphocytic lymphoma & small lymphocytic lymphoma | 982336, 959131 | 98233, 982336 | C911 | 2041 | 2041 |
| Diffuse large B-cell lymphoma | 96803 | 96833 | C833 |  |  |
| Hairy cell leukemia | 99403 | 99403 |  | 2024 |  |
| Mantle cell lymphoma | 96733 |  |  |  |  |
| Marginal zone lymphoma, mucosa-associated lymphoid tissue (MALT) | 96993 |  |  |  |  |
| Marginal zone lymphoma, not otherwise specified |  | 97113 |  |  |  |
| Precursor B-cell acute lymphoblastic leukemia | 98363 |  |  |  |  |
| Waldenstrom | 97613 | 97613, 96713 |  | 2092 | 2002, 2003 |

**Supplementary table S3**

SNOMED and ICD codes used to classify myeloproliferative neoplasms

| **Diagnosis** | **Morphologic code ICD-O/3** | **Morphologic code**  **ICD-O/2** | **ICD-10** | **ICD-9** | **ICD-8** | **ICD-7** |
| --- | --- | --- | --- | --- | --- | --- |
| Essential thrombocythemia (ET) | 99621, 99623 |  | D473 | 238H, 2079 | 207.1-9 | 2079 |
| Primary Myelofibrosis (PMF) | 99611 |  | D474 | 289W | 209 | 209 |
| Polycythemia Vera (PV) | 99501, 99503 |  | D459 | 238E/238.4  2091 | 208 | 208 |
| Myeloproliferative Neoplasm Unclassified (MPN-U) | 99601, 99751 |  | D471 | 238H |  |  |

**Supplementary table S4**

Occurrence of hematological malignancies +/- five years from the index date and > five years after the index date in individuals with SSc and matched comparators. Percentages are estimated within each group to assess if a higher proportion of malignancies present within +/- five years from index in individuals with SSc when compared to general population comparators

| **Hematological malignancies** | **Individuals with systemic sclerosis** | | | **Comparators** | | |
| --- | --- | --- | --- | --- | --- | --- |
|  | **+/- 5 years**  **(n, %)** | **> 5 years**  **(n, %)** | **Total** | **+/- 5 years**  **(n, %)** | **> 5 years**  **(n, %)** | **Total** |
| **Subgroup** |  |  |  |  |  |  |
| Lymphoid malignancies | 9 (50.0%) | 9 (50.0%) | 18 | 55 (58.5%) | 39 (41.5%) | 94 |
| Myeloid malignancies | 10 (76.9%) | 3 (23.1%) | 13 | 48 (56.5 %) | 37 (43.5 %) | 85 |
| Plasma cell malignancies | 3 (60.0%) | 2 (40.0%) | 5 | 17 (58.6%) | 12 (41.4%) | 29 |
| Cases that could not be classified as lymphoid or myeloid | 1 (50.0%) | 1 (50.0%) | 2 | 1 (100.0%) | 0 (0.0%) | 1 |
| **Myeloid malignancies** |  |  |  |  |  |  |
| Acute myeloid leukemia | 2 (100.0%) | 0 (0.0%) | 2 | 4 (44.4%) | 5 (55.5%) | 9 |
| Chronic myeloid leukemia | 1 (100.0%) | 0 (0.0%) | 1 | 6 (54.5%) | 5 (45.5%) | 11 |
| Myelodysplastic syndrome | 0 (0.0%) | 1 (100.0%) | 1 | 5 (50.0%) | 5 (50.0%) | 10 |
| Myeloid Leukemia not otherwise specified | 0 | 0 | 0 | 1 (100.0%) | 0 (0.0%) | 1 |
| **Myeloproliferative neoplasms** |  |  |  |  |  |  |
| Myeloproliferative neoplasms overall | 7 (77.8%) | 2 (22.2%) | 9 | 32 (59.3%) | 22 (41.7%) | 54 |
| Essential thrombocythemia | 4 (80.0%) | 1 (20.0%) | 5 | 11 (50.0%) | 11 (50.0%) | 22 |
| Myelofibrosis | 0 (0.0%) | 1 (100.0%) | 1 | 1 (50.0%) | 1 (50.0%) | 2 |
| Polycythemia vera | 2 (100.0%) | 0 (0.0%) | 2 | 14 (73.7%) | 5 (26.3%) | 19 |
| Myeloproliferative neoplasms unclassified | 1 (100.0%) | 0 (0.0%) | 1 | 6 (54.5%) | 5 (45.5%) | 11 |
| **Lymphoid malignancies** |  |  |  |  |  |  |
| B cell | 9 (50.0%) | 9 (50.0%) | 18 | 40 (54.1%) | 34 (45.9%) | 74 |
| T cell | 0 (%) | 0 (%) | 0 | 3 (50.0%) | 3 (50.0%) | 6 |
| Cases that could not be classified as B- or T-cell malignancies | 0 (%) | 0 (%) | 0 | 7 (77.8%) | 2 (22.2%) | 9 |
| **B-cell malignancies** |  |  |  |  |  |  |
| B-cell lymphoma not otherwise specified | 1 (100.0%) | 0 (0.0%) | 1 | 3 (42.9%) | 4 (57.1%) | 7 |
| Burkitt lymphoma | 1 (100.0%) | 0 (0.0%) | 1 | 0 (%) | 0 (%) | 0 |
| Chronic lymphocytic leukemia | 2 (40.0%) | 3 (60.0%) | 5 | 14 (56.0%) | 11 (44.0%) | 25 |
| Diffuse large B-cell lymphoma | 4 (66.7%) | 2 (33%) | 6 | 8 (61.5%) | 5 (38.5%) | 13 |
| Follicular lymphoma | 1 (33.3%) | 2 (66.7%) | 3 | 6 (54.5%) | 5 (45.5%) | 11 |
| Hairy cell leukemia | 0 (%) | 0 (%) | 0 | 0 (0.0%) | 1 (100.0%) | 1 |
| Marginal zone lymphoma | 0 (0.0%) | 2 (100.0%) | 2 | 4 (44.4%) | 5 (55.6%) | 9 |
| Mantle cell lymphoma | 0 (%) | 0 (%) | 0 | 1 (50.0%) | 1 (50.0%) | 2 |
| Waldenstrom | 0 (%) | 0 (%) | 0 | 2 (50.0%) | 2 (50.0%) | 4 |
| Precursor B-cell acute lymphoblastic leukemia | 0 (%) | 0 (%) | 0 | 2 (100.0%) | 0 (0.0%) | 2 |

**Supplementary table S5**

Hematological malignancies after index date. Crude incidence rates and rate differences of hematological malignancies overall, and in subgroups of hematological malignancies, in individuals with SSc and matched general population comparators. Hazard ratios for hematological malignancies comparing individuals with systemic sclerosis and comparators, adjusted for age and sex, as well as stratified on sex and age categories.

**Sensitivity Analysis.** Individuals who were diagnosed with any malignancies (solid or hematological) before the index date are excluded from this analysis.

|  | **Individuals with systemic sclerosis** | | | **Matched comparators** | | |  |  |  |  |  |
| --- | --- | --- | --- | --- | --- | --- | --- | --- | --- | --- | --- |
|  | **N events** | **Person-years** | **Crude IR**  **(95 % CI)**  *1000 person-years | **N events** | **Person- years** | **Crude IR**  **(95 % CI)**  *1000 person-years | **Crude rate difference**  *1000 person-years | **HR overall (95% CI)** | **HR 1 year** | **HR 5 years** | **HR 10 years** |
| **Overall** | 24 | 10 084 | 2.4 (1.6-3.5) | 129 | 114 856 | 1.3 (0.9 - 1.3) | 1.3 (0.3 - 2.2) | 2.3 (1.5 - 3.3) | 2.0 (1.0 – 4.2) | 2.2 (1.4 - 3.5) | 2.5 (1.3 - 4.8) |
| **Stratified by sex** | | | | | | | | |  |  |  |
| **Men** | 6 | 2 051 | 2.9 (1.3-6.5) | 30 | 24 282 | 1.2 (0.9 - 1.8) | 1.7 (-0.7 - 4.0) | 2.6 (1.0 - 5.2) | 3.3 (0.7 - 14.7) | 2.3 (0.9 - 6.0) | 2.2 (0.4 - 12.1) |
| **Women** | 18 | 8 032 | 2.2 (1.4 - 3.6) | 99 | 90 574 | 1.1 (0.9-1.3) | 1.1 (0.1 - 2.2) | 2.2 (1.3 - 3.4) | 1.8 (0.7 - 4.3) | 2.2 (1.3 - 3.7) | 2.6 (1.2 - 5.4) |
| **Age at index date, in groups** | | | | | | | | |  |  |  |
| **18 – 49** | 7 | 3 364 | 2.1 (1.0 - 4.4) | 11 | 35 862 | 0.3 (0.2 - 0.5) | 1.8 (0.2 - 3.3) | 6.9 (2.9 - 13.3) |  |  |  |
| **50 – 59** | 6 | 2 588 | 2.3 (1.0 - 5.2) | 30 | 27 586 | 1.1 (0.8 - 1.6) | 1.2 (-0.7 - 3.1) | 2.2 (0.9 - 4.4) |  |  |  |
| **60 – 69** | 3 | 2 514 | 1.2 (0.4 - 3.7) | 42 | 30 051 | 1.4 (1.0 - 1.9) | -0.2 (-1.6 - 1.2) | 0.9 (0.2 - 2.3) |  |  |  |
| **>70** | 8 | 1 617 | 4.9 (2.5 - 9.9) | 46 | 21 354 | 2.1 (1.6 - 2.9) | 2.8 (-0.7 - 6.3) | 2.3 (1.1-4.3) |  |  |  |
| **Subgroups of hematological malignancies** | | | | | | | | |  |  |  |
| **Lymphoid malignancies** | 15 | 10 101 | 1.5 (0.9 - 2.5) | 63 | 115 072 | 0.5 (0.4 - 0.7) | 0.9 (0.2 - 1.7) | 2.9 (1.4 - 3.9) | 2.1 (0.7- 6.0) | 2.5 (1.4 - 4.6) | 4.0 (1.8 - 8.7) |
| **B-cell malignancies** | 15 | 10 101 | 1.5 (0.9 - 2.5) | 43 | 115 098 | 0.5 (0.3 - 0.6) | 1.0 (0.3 - 1.8) | 3.5 (2.0 – 5.6) | 2.7 (0.9 - 8.0) | 3.0 (1.6 - 5.6) | 4.4 (2.0 - 9.7) |
| **Myeloid**  **malignancies** | 7 | 10 127 | 0.7 (0.3 - 1.4) | 43 | 115 199 | 0.4 (0.3 - 0.5) | 0.3 (-0.2 - 0.8) | 2.0 (0.9 - 3.9) | 2.5 (0.7 - 8.8) | 1.9 (0.8 - 4.5) | 1.6 (0.4 - 5.9) |
| **Plasma cell malignancies** | 2 | 10 137 | 0.2 (0.0 - 0.8) | 24 | 115 274 | 0.2 (0.1 - 0.3) | 0.0 (-0.3 - 0.3) | 1.0 (0.2 – 3.0) |  |  |  |

N = number of observations, IR = incidence rate, CI = confidence interval, HR = hazard ratio

**Supplementary table S6**

Hematological malignancies after index date. Crude incidence rates and rate differences of hematological malignancies overall, and in subgroups of hematological malignancies, in individuals with SSc and matched general population comparators. Hazard ratios for hematological malignancies comparing individuals with systemic sclerosis and comparators, adjusted for age and sex, as well as stratified on sex and age categories.

**Sensitivity Analysis.** Individuals who were diagnosed with any solid malignancy before the index date are excluded from this analysis. Additionally, individuals have been censored when having a **solid cancer after the index date**, at the time of solid cancer.

|  | **Individuals with systemic sclerosis** | | | **Matched comparators** | | |  |  |  |  |  |
| --- | --- | --- | --- | --- | --- | --- | --- | --- | --- | --- | --- |
|  | **N**  **events** | **Person - years** | **Crude IR**  **(95% CI)**  ***1000 person-years** | **N**  **events** | **Person - years** | **Crude IR**  **(95% CI)**  ***1000 person-years** | **Crude rate difference**  ***1000 person-years** | **HR overall**  **(95% CI)** | **HR 1 year** | **HR 5 years** | **HR 10 years** |
| **Overall** | 23 | 9 439 | 2.4 (1.6 - 3.7) | 118 | 109 288 | 1.1 (0.9 - 1.3) | 1.4 (0.3 - 2.4) | 2.4 (1.6 - 3.6) | 2.0 (0.9 - 4.2) | 2.5 (1.6 - 3.9) | 3.0 (1.5 – 6.0) |
| **Stratified by sex** | | | | | | | | | | | |
| **Men** | 6 | 1 837 | 3.3 (1.5 - 7.3) | 27 | 22 798 | 1.2 (0.8 - 1.7) | 2.1 (-0.6 - 4.7) | 3.1 (1.2 - 6.0) | 3.0 (0.6 - 13.9) | 2.6 (1.0 - 6.6) | 3.6 (0.7 - 19.9) |
| **Women** | 17 | 7 601 | 2.2 (1.4 - 3.6) | 91 | 86 490 | 1.1 (0.9 - 1.3) | 1.2 (0.1 - 2.3) | 2.3 (1.3 - 3.6) | 1.7 (0.7 - 4.3) | 2.4 (1.4 - 4.0) | 2.9 (1.4 - 6.3) |
| **Age at index date in categories** | | | | | | | | | | | |
| **18-49** | 7 | 3 263 | 2.15 (1.0 - 4.5) | 11 | 34 984 | 0.3 (0.2 - 0.6) | 1.8 (0.2 - 3.4) | 6.9 (3.0 - 13.4) |  |  |  |
| **50-59** | 6 | 2 446 | 2.4 (1.1 - 5.5) | 26 | 26 396 | 1.0 (0.7 - 1.5) | 1.5 (-0.5 - 3.5) | 2.5 (1.0 - 5.1) |  |  |  |
| **60-69** | 3 | 2 277 | 1.3 (0.4 - 4.1) | 39 | 28 141 | 1.4 (1.0 - 1.9) | -0.1 (-1.6 - 1.5) | 1.0 (0.2 - 2.5) |  |  |  |
| **> 70** | 7 | 1 453 | 4.8 (2.3 -10.1) | 42 | 19 767 | 2.1 (1.6 - 2.9) | 2.7 (-0.9 - 6.3) | 2.3 (1.0 - 4.5) |  |  |  |
| **Subgroups of hematological malignancies** | | | | | | | | | | | |
| **Lymphoid malignancies** | 15 | 9 485 | 1.6 (1.0 - 2.6) | 54 | 109 716 | 0.5 (0.4 - 0.6) | 1.1 (0.3 - 1.9) | 3.5 (2.0 - 5.6) | 2.1 (0.7 - 6.2) | 3.1 (1.7 - 5.7) | 5.5 (2.4 - 12.5) |
| **B-cell malignancies** | 15 | 9 485 | 1.6 (0.9 - 2.6) | 45 | 109 872 | 0.4 (0.3 - 0.6) | 1.2 (0.4 – 2.0) | 4.3 (2.5 - 6.8) | 2.8 (0.9 - 8.5) | 3.8 (2.0 – 7.0) | 6.0 (2.6 – 14.0) |
| **Myeloid malignancies** | 6 | 9 497 | 0.6 (0.3 - 1.4) | 41 | 109 867 | 0.4 (0.3 - 0.5) | 0.3 (-0.3 – 0.8) | 1.9 (0.7 - 3.7) | 2.1 (0.6 - 7.9) | 1.8 (0.7 - 4.4) | 1.6 (0.4 - 6.3) |
| **Plasma cell malignancies** | 3 | 9 534 | 0.3 (0.1 – 1.0) | 24 | 110 124 | 0.2 (0.2 - 0.3) | 0.1 (-0.3 - 0.5) | 1.6 (0.4 – 4.0) |  |  |  |

|  | **Individuals with systemic sclerosis** | | | **Matched comparators** | | |  |  |  |  |  |
| --- | --- | --- | --- | --- | --- | --- | --- | --- | --- | --- | --- |
|  | **N**  **events** | **Person**  **- years** | **Crude IR**  **(95% CI)**  ***1000 person-years** | **N**  **events** | **Person**  **- years** | **Crude IR**  **(95% CI)**  ***1000 person-years** | **Crude rate difference**  ***1000 person-years** | **HR overall**  **(95% CI)** | **HR 1 year** | **HR 5 years** | **HR 10 years** |
| **Overall** | 23 | 10 101 | 2.3 (1.5 - 3.4) | 154 | 130 723 | 1.2 (1.0 - 1.4) | 1.1 (0.2 - 2.1) | 2.1 (1.4 - 3.1) | 2 (1.0 - 4.1) | 2.1 (1.3 - 3.3) | 2.2 (1.1 - 4.4) |
| **Stratified by sex** | | | | | | | | | | | |
| **Men** | 7 | 2 098 | 3.3 (1.6 - 7.0) | 34 | 26 151 | 1.3 (0.9 - 1.8) | 2.03 (-0.5 -4.5) | 2.8 (1.2 - 5.5) | 3.4 (0.8 - 14.9) | 2.3 (0.9 - 5.6) | 3.1 (0.8 -12.0) |
| **Women** | 16 | 8 002 | 2.0 (1.2 - 3.3) | 120 | 104 572 | 1.2 (1.0 - 1.4) | 0.9 (-0.2 - 1.9) | 1.9 (1.1 - 3.0) | 1.7 (0.7 - 4.2) | 1.9 (1.1 - 3.3) | 2.1 (0.9 - 4.7) |
| **Age at index date in categories** | | | | | | | | | | | |
| **18-49** | 6 | 3 323 | 1.8 (0.8 - 4.0) | 11 | 37 841 | 0.3 (0.2 - 0.5) | 1.5 (0.1 – 3.0) | 6.3 (2.5 - 12.8) |  |  |  |
| **50-59** | 6 | 2 516 | 2.4 (1.1 - 5.3) | 32 | 30 828 | 1.0 (0.7 - 1.5) | 1.4 (-0.6 - 3.3) | 2.4 (0.9 - 4.8) |  |  |  |
| **60-69** | 3 | 2 534 | 1.2 (0.4 - 3.7) | 51 | 35 205 | 1.5 (1.1 - 1.9) | -0.3 (-1.7 - 1.1) | 0.8 (0.2 - 2.2) |  |  |  |
| **> 70** | 8 | 1 729 | 4.6 (2.3 - 9.3) | 60 | 26 849 | 2.2 (1.7 - 2.9) | 2.4 (-0.9 - 5.7) | 2.1 (1.0 - 3.9) |  |  |  |
| **Subgroups of hematological malignancies** | | | | | | | | | | | |
| **Lymphoid malignancies** | 13 | 10 157 | 1.3 (0.7 - 2.2) | 75 | 131 323 | 0.6 (0.5 - 0.7) | 0.7 (0 - 1.4) | 2.5 (1.4 - 4.1) | 2.0 (0.7 – 6.0) | 2.1 (1.1 - 3.9) | 3.3 (1.4 - 7.8) |
| **B-cell malignancies** | 13 | 10 169 | 1.3 (0.7 - 2.2) | 62 | 131 507 | 0.5 (0.4 - 0.6) | 0.8 (0.1 - 1.5) | 3.0 (1.7 – 5.0) | 2.7 (0.9 – 8.0) | 2.6 (1.3 - 4.9) | 3.7 (1.6 - 8.9) |
| **Myeloid malignancies** | 7 | 10 162 | 0.7 (0.3 - 1.4) | 57 | 131 492 | 0.4 (0.3 - 0.6) | 0.3 (-0.3 - 0.8) | 1.7 (0.8 - 3.4) | 2.2 (0.7 - 7.3) | 1.6 (0.7 - 3.9) | 1.3 (0.3 - 5.1) |
| **Plasma cell malignancies** | 4 | 10 206 | 0.4 (0.2 - 1.0) | 25 | 131 863 | 0.2 (0.1 - 0.3) | 0.2 (-0.2 - 0.6) | 2.2 (0.7 - 5.2) |  |  |  |

**Supplementary table S7**  Hematological malignancies after index date. Crude incidence rates and rate differences of hematological malignancies overall, and in subgroups of hematological malignancies, in individuals with SSc and matched general population comparators. Hazard ratios for hematological malignancies comparing individuals with systemic sclerosis and comparators, adjusted for age and sex, as well as stratified on sex and age categories. **Sensitivity analysis.** Individuals with an ICD-10 code indicating Sjögren’s disease (M35.0, M35.0A, M35.0B) prior to the index date were excluded. Individuals with an ICD-10 code indicating Sjögren’s disease after the index date resulted in censoring of the observation.
